# Transcription factories

**DOI:** 10.3389/fgene.2012.00221

**Published:** 2012-10-23

**Authors:** Dietmar Rieder, Zlatko Trajanoski, James G. McNally

**Affiliations:** ^1^Division of Bioinformatics, Biocenter, Innsbruck Medical UniversityInnsbruck, Austria; ^2^Laboratory of Receptor Biology and Gene Expression, National Cancer Institute, National Institutes of HealthBethesda, MD, USA

**Keywords:** transcription factories, RNA polymerase II, nucleus, gene clustering, transcription, factory

## Abstract

There is considerable evidence that transcription does not occur homogeneously or diffusely throughout the nucleus, but rather at a number of specialized, discrete sites termed transcription factories. The factories are composed of ~4–30 RNA polymerase molecules, and are associated with many other molecules involved in transcriptional activation and mRNA processing. Some data suggest that the polymerase molecules within a factory remain stationary relative to the transcribed DNA, which is thought to be reeled through the factory site. There is also some evidence that transcription factories could help organize chromatin and nuclear structure, contributing to both the formation of chromatin loops and the clustering of active and co-regulated genes.

## INTRODUCTION

Cellular processes like differentiation or the response to physiological stimuli all require coordinated and efficient regulation of many genes. This regulation can now be monitored at thousands of genes simultaneously using high throughput microarrays and next-generation sequencing. These technologies have revealed temporally coordinated changes in the transcription levels of many genes in response to developmental or environmental changes. However, in addition to this temporal coordination of gene transcription, there is strong experimental evidence that transcription is also spatially coordinated within each cell nucleus. Specifically, it is now recognized that transcription occurs at discrete foci located throughout the nucleus. Here we review first the data supporting this observation, and then discuss its potential consequence for polymerase function, gene regulation, and nuclear organization.

## RNA POLYMERASE CLUSTERING

**FIGURE 1:**
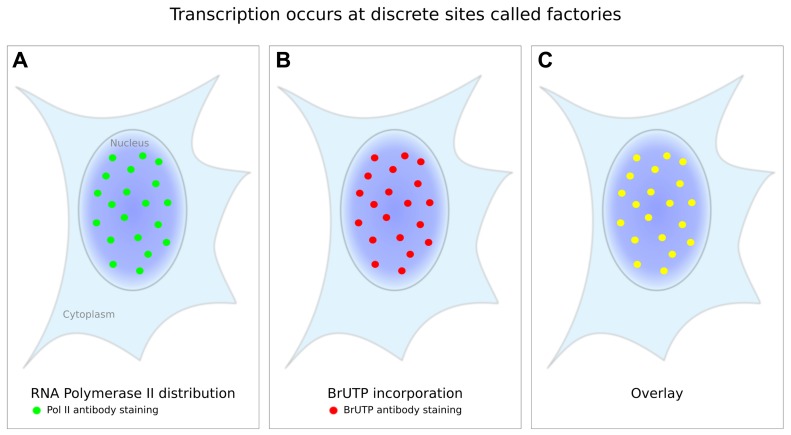
**Gene transcription occurs at discrete sites in the nucleus. (A)** When RNA polymerase II is detected by immunofluorescence a non-uniform staining pattern can be observed (green dots). **(B)** Labeling of nascent RNA by Br-UTP incorporation and subsequent immuno-staining (red dots) reveals a staining pattern that matches the polymerase staining as an overlay **(C)** shows (yellow dots). These discrete sites of active transcription are referred to as “transcription factories”. The number of factories per nucleus varies from ~100–8,000 depending on the cell type, differentiation state, and the detection method.

The term transcription “factory” was first used in 1993 by Jackson and colleagues. They exploited Br-UTP labeling to visualize mRNA synthesis in permeabilized HeLa cells that were encapsulated in agarose microbeads. Using confocal microscopy to visualize the transcripts, they found that transcription occurred at 300–500 discrete sites in the nucleus (Jackson et al., 1993), rather than being homogeneously distributed throughout the nucleus. Similar results were obtained by Wansink et al. (1993) using the same methods, namely Br-UTP labeling of nascent RNA detected by immunofluorescence microscopy. These initial studies were further supported by Iborra et al. (1996) who used either Br-UTP or biotin-14 CTP to pulse-label nascent transcripts, and who then visualized the transcripts not only by light microscopy but also by electron microscopy using immuno-gold particles. Once again, this yielded a discrete labeling pattern rather than a homogeneous distribution throughout the nucleus. Iborra et al. (1996) found that the signal increased in these distinct transcript clusters with the duration of labeling (1, 5, and 15 min), suggesting that the clusters marked active synthetic sites. However, the total number of clusters per cell did not increase with longer labeling. This suggests the method is sensitive enough to detect all sites, otherwise longer labeling should have revealed additional sites. Furthermore, it suggests that all incorporation, i.e., transcription, occurs in the clusters (**Figure 1**), which are now commonly referred to as transcription factories.

To further investigate transcription factories, Iborra et al. (1996) compared the distribution of transcripts with the distribution of RNA polymerase II. Using transmission electron microscopy, they labeled both nascent transcripts and polymerase molecules with gold particles of different sizes (9 and 15 nm, respectively) and identified two populations of labeled polymerase molecules: a background of scattered lone particles and a second population organized in clusters. The scattered lone polymerase molecules did not co-localize with transcripts, whereas the clustered polymerase molecules showed a one-to-one co-localization with transcripts, suggesting that the clusters contain transcriptionally engaged polymerase. The transcript and polymerase clusters were partially overlapping, with their centers separated by an average distance of ~24 nm. This co-localization of RNA polymerase II sites and nascent transcript has been confirmed by light microscopy in many succeeding studies (e.g., Grande et al., 1997; Osborne et al., 2004).

It is interesting to note that these analyses of transcription factories were mirrored by parallel studies of DNA replication using biotin-11 UTP or Br-UTP. Like RNA synthesis, these studies demonstrated that DNA synthesis occurred at discrete sites throughout the nucleus (Nakamura et al., 1986; Nakayasu and Berezney, 1989; Hozák et al., 1993) leading to the analogous concept of replication factories.

Although transcription factories are easily visualized in fixed cells by immunofluorescence either by electron microscopy or confocal light microscopy, they are not easily visualized in live eukaryotic cells by GFP tagging. Rather, in such cells, a diffuse nuclear fluorescence is typically seen. Hieda et al. (2005) have argued that this arises for two reasons. First, confocal sections may be too thick to distinguish the multiple factories that are expected to be found within a single section. Consistent with this possibility, GFP-tagged polymerase clusters have in fact been seen *in vivo* in much thinner bacterial cells (Jin and Cabrera, 2006). Second, there are on average only ~8 polymerase molecules per factory (see below), which would make it difficult to detect a signal from a single factory without some form of signal amplification such as immunofluorescence. Consistent with this possibility is the observation that a cluster of polymerase molecules has been observed in live and fixed cells at the site of a tandem gene array (Becker et al., 2002; Müller et al., 2007), which given its strong levels of transcription might be expected to require a factory with many more polymerase molecules. In addition, it has been recently shown that the polymerase factories in the transformed cell lines that are typically used for live cell imaging are significantly smaller (corresponding presumably to fewer polymerase molecules per factory) than those used in some of the fixed cell transcription factory analyses, such as mouse erythroblasts (Eskiw and Fraser, 2011). In sum, given the limited examples of transcription factories in live cells, more work is needed to visualize individual factories in live eukaryotic cells. This could be done using the most sensitive detectors and cameras now available, combined with various super-resolution approaches (Hell and Wichmann, 1994; Donnert et al., 2006; Tokunaga et al., 2008; Schermelleh et al., 2010). With these approaches, direct comparison of live and fixed cells of the same type and developmental state would be valuable.

While most work on transcription factories has been done based on RNA polymerase II, the other two RNA polymerases in the nucleus, namely RNA polymerase I and III, are also found in distinct foci that have likewise been identified as factories. RNA polymerase I specializes in the production of 45S rRNA and distinct foci of polymerase I with clusters of associated rRNA transcripts are found within nucleoli as determined by immuno-electron microscopy and fluorescence *in situ* hybridization (FISH; Hozák et al., 1994; Mosgoeller et al., 2001). Similarly, immuno-electron microscopy experiments showed that RNA polymerase III, which is responsible for the synthesis of 5S rRNA and tRNAs, is also found in clusters that are associated with clusters of 5S rRNA and tRNA transcripts (Pombo et al., 1999). In the remaining paragraphs of this article, we will focus on RNA polymerase II where most of the work on factory analysis has been performed.

## TRANSCRIPTION FACTORY STRUCTURE

Estimates of the number of polymerase molecules in a single transcription factory range from 4 to 30 dependent on the technique and cell type used. The lower bound estimate of four comes from measurements of the number of RNA molecules in a factory that were protected from RNase A digestion, presumably reflecting only those molecules shielded by direct association with the polymerase in the act of transcription (Iborra et al., 1996). The upper bound estimate of 30 comes from estimating ~75,000 nascent transcripts (which was measured by extending truncated transcripts in permeabilized cells in the presence of [^32^P]uridine triphosphate) combined with counting ~2,400 transcription foci (which were detected by electron microscopy) yielding 75,000/2,400 ≈ 30 polymerase molecules per factory (Jackson et al., 1998). A similar calculation based on unpublished data is cited by Cook et al. who estimate ~65,000 active polymerase molecules and ~8,000 polymerase foci, yielding 65,000/8,000 ≈ 8 polymerase molecules per factory (Martin and Pombo, 2003). These three different estimates suggest that most factories are not large conglomerates of polymerase molecules but rather contain 10 or so polymerase molecules.

Initially, transcription factories were thought to be ~71 nm in diameter (Iborra et al., 1996). This was determined by measuring the average diameter of active transcription sites in HeLa cells marked by clusters of immuno-gold particles in electron microscopy. A subsequent measurement in HeLa cells yielded a somewhat larger value of ~87 nm, with a spread ranging from 40 to 180 nm (Eskiw et al., 2008). These later measurements were done with energy filtering transmission electron microscopy (EFTEM) which was used to identify a nitrogen-rich proteinaceous core associated with Br-UTP incorporation (Eskiw et al., 2008). The diameter of this core was measured, since it presumingly reflects the cluster of polymerase molecules. More recently, EFTEM has been used again to measure factory diameters, but now in *ex vivo* murine fetal liver erythroblasts where a mean factory diameter of ~130 nm was measured (Eskiw and Fraser, 2011). In the same study, EFTEM was coupled with immuno-electron microscopy yielding a mean diameter of ~174 nm for factories that were enriched in the transcription factor KLF1. Finally, the same study also used EFTEM coupled with electron-microscopy *in situ* hybridization to identify factories associated with the highly active globin genes. These factories were larger still, measuring on average ~198 nm (Eskiw and Fraser, 2011). Together these data suggest that transcription factory size may depend on the transcriptional activity of the genes within the factory, with more transcriptionally active loci recruiting more polymerase leading to larger foci. In addition, the data suggest that there may be differences in factory size between transformed and non-transformed cells, although this could at least in part also reflect differences in transcriptional activity.

**FIGURE 2:**
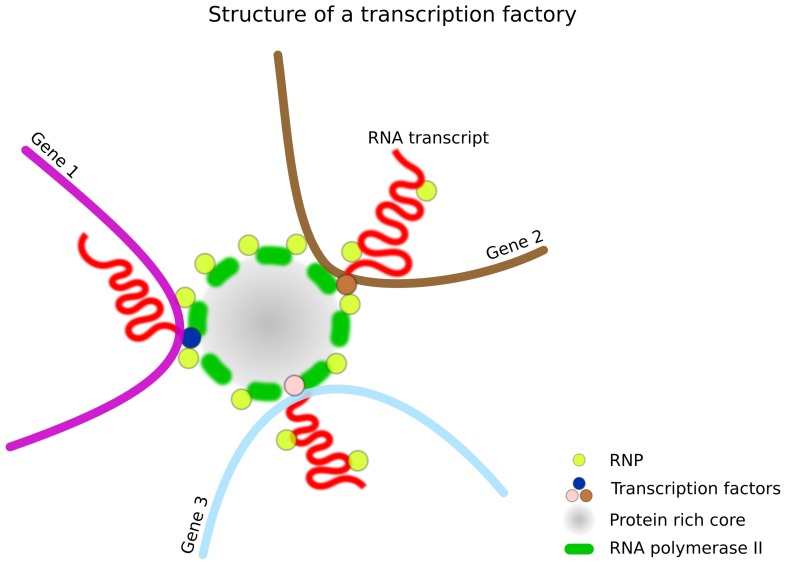
**Structure of a transcription factory.** Each factory contains 4–30 stationary RNA polymerase II molecules which are located on the surface of a protein-rich core (87 nm in diameter, as determined by EFTEM in HeLa cells). These proteins include many factors involved in transcription such as co-activators, chromatin remodelers, transcription factors, histone modification enzymes, RNPs, RNA helicases, and splicing and processing factors. Multiple genes can be processed by the same factory (three are shown). The size of a factory varies from 40 to 198 nm depending on the cell type, factory type, and detection or measurement method.

High-resolution microscopy has also started to provide information about the ultrastructure of transcription factories. Electron spectroscopic imaging permits the detection of nitrogen and phosphorus distributions at electron microscopy resolution, which can be used to infer the distribution of protein and nucleic acid in a structure (Eskiw et al., 2008). When applied to HeLa cells the method revealed that transcription factories contain a protein-rich porous core. The core diameters were found to be normally distributed around a mean of 87 nm, with diameters of 75% of the cores lying between 60 and 120 nm (Eskiw et al., 2008), but with some outliers ranging from <40 to >180 nm. The authors speculate that this protein-rich core has RNA polymerase II attached to the surface, and contains some or all of the many different proteins involved in making a mature transcript (**Figure 2**). The spectroscopic images detect very few phosphorus atoms (marking the backbone of nucleic acids) in the proteinaceous core, suggesting that the DNA template and the nascent transcript must lie outside of the core, presumably attached to the surface as well.

In addition to RNA polymerase and mRNA, transcription factories appear to contain virtually all of the other molecules that one might expect to find at a transcription site. The molecular composition of factories was determined by isolating factory components and then performing mass spectrometry. Factory components were isolated by subjecting cells to permeabilization and DNaseI digestion, which had previously been shown to leave the polymerase factories in an insoluble fraction (Jackson et al., 1988, 1993). Factory components were then released from this fraction by protease (i.e., caspases) digestion. The isolated components were shown to contain a substantial fraction of nascent mRNA and they also retained considerable polymerase activity as demonstrated by a run-on assay (Melnik et al., 2011). These factory isolates were then subjected to mass spectrometry, which revealed the presence of various transcription regulators such as co-activators and chromatin remodelers, transcription factors, histone modification enzymes, RNPs, splicing and processing factors, RNA helicases, and many more (Melnik et al., 2011).

The number of transcription factories in a nucleus appears to depend on the cell type and species, and also in part on the measurement procedure. For example, in cultured mouse embryonic fibroblasts ~1500 transcription factories have been detected by immunofluorescence of active RNA polymerase II (Osborne et al., 2004), whereas in cells taken directly from various mouse tissues the same group found from ~100 to 300 transcription factories dependent on the tissue type (Osborne et al., 2004). Even in similar cell types, different numbers have been reported in different species. For example, ~100–550 transcription factories have been detected in mouse erythroid cells (Osborne et al., 2004; Eskiw and Fraser, 2011), whereas ~1500 have been reported in human erythroblasts (Brown et al., 2008). Beyond differences in cell type, these estimates can also be influenced by the measurement procedure itself. The number of factories found in HeLa cells varies considerably depending on the imaging procedure: 300–500 factories by conventional fluorescence microscopy (Jackson et al., 1993); 2100 factories by combining electron and confocal microscopy (Iborra et al., 1996); 849–3,888 factories by 3D deconvolution microscopy (Fay et al., 1997); and ~10,000 factories (8,000 RNA polymerase II; 2,000 RNA polymerase III) by cryo-sectioning (Pombo et al., 1999). In sum, most data would suggest that factory numbers range from a few hundred to a thousand per nucleus, with perhaps some exceptional cases in which factory numbers may be much larger.

## ASSEMBLY AND MAINTENANCE OF TRANSCRIPTION FACTORIES

There is conflicting evidence about whether transcription factories can assemble *de novo* in response to transcriptional demands, or whether they are relatively stable structures whose number in a cell nucleus remains relatively constant.

Over the short term, the number of transcription factories in a nucleus remains fixed. During pulse-labeling of mRNA over a time span of 15 min no new labeled spots were observed at later time points, indicating that new transcription factories did not emerge at later times (Iborra et al., 1996). This is consistent with a model of pre-assembled factories. Further indication for pre-assembled factories comes from a study (Ferrai et al., 2010) which identified two different types of factories: one contained active (phosphorylated at Ser5 and Ser2) and the other poised RNA polymerase II (phosphorylated at Ser5 only). The poised factories were associated with the inducible *uPA* gene prior to its activation (Ferrai et al., 2010), suggesting that the factory was pre-assembled. Evidence that assembled factories are stably maintained in the absence of transcription is provided by a study (Mitchell and Fraser, 2008) in which transcription was inhibited, by either 5,6-dichlorobenzimidazole 1-β-D-ribofuranoside (DRB) to inhibit elongation (Chodosh et al., 1989; Marshall and Price, 1992) or heat shock to globally turn off transcription (Lindquist, 1986; Lis and Wu, 1993). During both treatments the number of factories per cell did not change and RNA polymerase II was still distributed in localized foci. These data suggest that transcription factories can exist in the absence of transcription and that they remain unchanged when transcription is abruptly halted. However, closer inspection of the western blot data in Mitchell and Fraser (2008) suggest that the number of polymerase molecules in a factory may decrease over time after transcription is inhibited, as the published data suggest that after transcriptional inhibition the fraction of active (defined as the immobilized/insoluble) polymerase is decreased while the fraction of the inactive (soluble) form is increased (see Figure 2 in Mitchell and Fraser, 2008). This raises the possibility that polymerase molecules are gradually released from factories in the absence of transcription and could potentially disappear altogether after an extended period of inhibition.

In contrast to the preceding observations, results obtained from live cell studies using GFP-tagged RNA polymerase II have suggested that transcription factories are dynamic and so in principle could assemble or disassemble on demand. The direct evidence for this comes from studies of two different artificial tandem array systems, where the loci in question can be identified in live cells by fluorescence tags that localize to the arrays. In both systems, transcriptional induction leads to an increase in GFP polymerase fluorescence at the array site (Becker et al., 2002; Darzacq et al., 2007). This suggests recruitment of the polymerase to the gene array and formation of a factory there, rather than migration of the activated gene array to a pre-existing factory. Similar results were obtained in *Drosophila* polytene nuclei, where freely diffusing GFP-tagged RNA polymerase II molecules appeared to be recruited to the *Hsp70* locus upon heat shock (Yao et al., 2007). Note, however, there are several caveats to these interpretations. First, all three of these systems are unusual, and may not reflect transcription at normal single copy genes. Second, it is possible that if cells have a large number of factories, then relocation to the nearest factory could occur over a distance too short to be detected by conventional light microscopy. Third, it is also possible that in each of these systems, genes were already in contact with a small factory due to basal transcription levels, and that the increased transcriptional load upon induction led to a recruitment of more polymerase molecules creating the false impression that a factory was created at the site. Note this latter possibility is consistent with the increased size of KLF1-enriched factories and the factories engaged with highly expressed genes observed in mouse erythroid cells (Eskiw and Fraser, 2011).

Another set of live cell studies, namely fluorescence recovery after photobleaching (FRAP), also addresses the question of whether factories are stable or dynamic structures (Becker et al., 2002; Kimura et al., 2002; Hieda et al., 2005). Consistent with the notion of a stable structure, FRAPs of the polymerase typically show a very slow component (~25% of the total measured intensity) that takes on the order of 10–20 min for full recovery. However, evidence suggests that this slow phase is associated with the elongation state of the polymerase, since it disappears when cells are treated with DRB, which prevents entry of the polymerase into elongation. Interestingly, after DRB treatment, FRAP of the polymerase then requires only a few seconds for complete recovery. If the polymerase were stably bound to a nuclear substructure, then FRAPs of the polymerase after DRB treatment should reveal this stable binding. The absence of such a residual slow component suggests that factories may be like many other nuclear structures, including Cajal bodies and speckles, where the constituents are in constant flux leading nevertheless to a visible structure in steady state (Dundr and Misteli, 2010; Dundr, 2012).

## TRACKING OR STATIONARY POLYMERASES WITHIN A FACTORY?

The conventional view is that the comparatively small RNA polymerase molecule travels down the giant DNA polymer. *In vitro* experiments have provided support for this view by visualizing single prokaryotic RNA polymerase molecules sliding along immobilized stretches of DNA (Kabata et al., 1993; Harada et al., 1999). However, studies in which the polymerase was attached to a surface have demonstrated that RNA polymerase can rotate the DNA template and thread it through the protein as it is transcribed (Wang et al., 1998; Guthold et al., 1999). These single molecule experiments have indicated that RNA polymerase is a powerful molecular motor (Herbert et al., 2008), but which part is moving depends on which one is fixed – either the template or the enzyme – so *in vitro* experiments under artificial conditions may not necessarily reflect the situation in a living cell.

Some evidence suggests that in live cells it is the polymerase that is stationary. The first suggestion of this came from the identification of transcription factory sites based on Br-UTP incorporation into mRNA observed at the electron microscopy level. Here it was found that the diameter of the mRNA clusters, measuring ~75 nm, remained constant although the transcript length increased during synthesis. Even over an extended chase period, during which the transcripts grew from ~34 bp (at 1****min) to ~2,070 bp (at 30 min), they still occupied the same small volume (Iborra et al., 1996). This volume would be expected to increase in proportion to the length of the transcribed template if the polymerase were tracking along the DNA template and thereby moving away from the initial site of transcription along with the nascent RNA.

**FIGURE 3:**
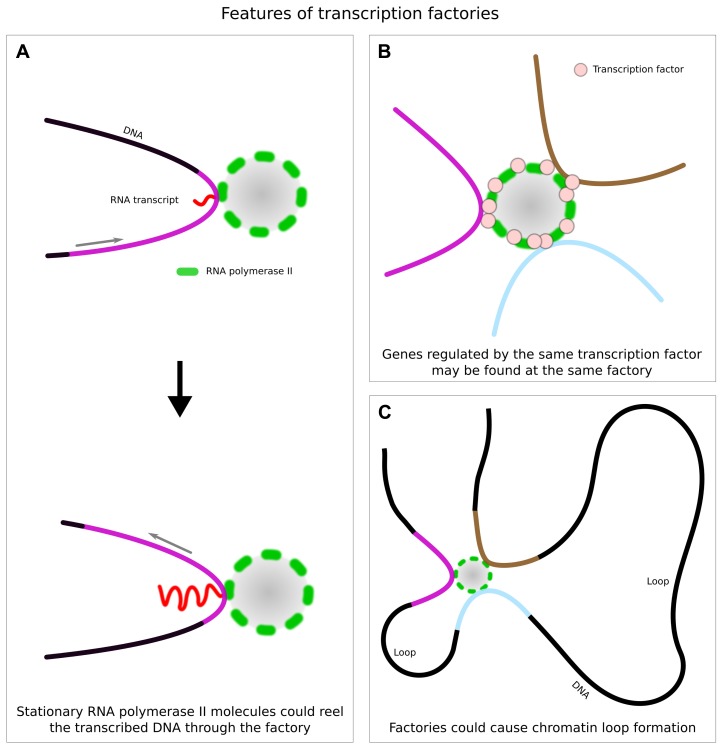
**Features of transcription factories. (A)** Some evidence suggests that factories are stationary and that the transcribed DNA template is reeled through the factory (small gray arrows) while the resulting RNA transcript is extruded. **(B)** Some experimental evidence supports the model of “specialized” or “preferred” transcription factories, where a given transcription factor (pink dot) is enriched and multiple genes regulated by this factor are transcribed. **(C)** Genes that are located on the same chromosome and transcribed by the same factory could cause formation of chromatin loops at the transcription site.

Additional evidence in support of a stationary polymerase comes from Papantonis et al. (2010). They examined two genes that could both be switched on rapidly by addition of TNFα. One gene was short (11 kb) and so was transcribed multiple times after induction, while the other gene was long (221 kb) and so required ~75 min for one round of transcription. The authors used chromosome conformation capture 3C (Dekker et al., 2002), which identifies DNA sequences that lie close together in the nuclear volume, to monitor the relative positions of these two genes. 3C analysis of the two TNFα-regulated genes showed that they were far apart before induction, but immediately after induction the two promoter sequences were in close proximity. This suggests that the two genes are recruited to the same polymerase factory. Then they repeated these proximity measurements at 10, 30, 60, and 85 min after induction and with later time intervals observed that progressively more downstream regions of the long gene were in close proximity to the short gene. These 3C data were confirmed by RNA FISH and super-resolution microscopy, which showed that over time transcripts from the short gene co-localized with transcripts from more and more downstream regions of the long gene. These observations are consistent with a model in which the two genes are recruited to the same polymerase factory with the corresponding DNA sequences reeled through stationary polymerases in that factory (**Figure 3A**).

Another piece of indirect evidence supporting the stationary polymerase model comes from an immunofluorescence analysis of polymerase factories, nascent transcripts and histone modifications at a tandem gene array (Müller et al., 2007). Here it was found that nascent transcripts from the genes in the array co-localized with the polymerase factories, however, a histone mark for recently transcribed DNA was not co-localized with the factories or the transcripts, but rather distributed in a much larger peripheral zone surrounding the factories and their transcripts. Thus recently transcribed DNA appeared to be extruded from the factory site, as would be expected if the polymerase was stationary and reeled the DNA template through it.

These preceding studies are all consistent with a stationary polymerase but more direct *in vivo* evidence will be required to demonstrate convincingly that the DNA of transcribed genes is actually moving relative to the factories. It also remains to be investigated how a stationary polymerase can transcribe genes on the sense and antisense strand of the same region at the same time. In such a “tug-of-war” scenario the DNA would be pulled in opposite directions simultaneously, thereby potentially abrogating any movement.

Another missing piece of evidence in the stationary polymerase model is how the polymerase is tethered and what it is tethered to in order to remain stationary. Several lines of evidence suggest that the polymerase could be associated with a nuclear matrix (Jackson et al., 1981, 1984b, 1990; Jackson and Cook, 1985; Jackson, 1997; Gavrilov et al., 2010). Jackson and Cook (1985) found that after removing restriction-enzyme-digested chromatin by electroelution from agarose-embedded nuclei, more than 90% of nascent RNA, their templates and polymerases remained in the nuclei. In addition, the transcription complexes retained most of their activity and the DNA retained in the nucleus was enriched in sequences of actively transcribed genes. This led to the conclusion that the transcription machinery is tied to a structure that was not affected by restriction enzymes and therefore could not be eluted from nuclei. Using Matrix 3C (M3C), a variant of 3C which includes a high salt nuclear extraction and subsequent analysis of matrix-bound ligation products, Gavrilov et al. (2010) found that multiple genes were associated at a transcription factory that was stabilized by the nuclear matrix. Moreover, a proteomic analysis focusing on large fragments of transcription factories revealed some structural components, including spectrins, lamins, and actin (Melnik et al., 2011), that could be involved in securing transcription factories. Consistent with the proteomic identification of lamins as component of transcription factories a study using RNAi knockdown of lamins found that lamin-B1 is required for the assembly of active sites of RNA synthesis. Decreased RNA synthesis was observed when lamin-B1 was depleted and it was correlated with a massive change in the overall nuclear structure and organization of chromosome territories (Tang et al., 2008). Despite this evidence, the existence of a nuclear matrix or “nucleoskeleton” is still hotly debated (Pederson, 2000; Misteli, 2007), in large part because this structure has not been directly visualized in either live or fixed cells that have not been subjected to extraction procedures.

## TRANSCRIPTION FACTORIES AS ORGANIZERS OF NUCLEAR STRUCTURE

Factories have been suggested as a key organizing principle for both nuclear and chromatin structure (Cook, 2002; Marenduzzo et al., 2007; Sexton et al., 2007). In terms of nuclear structure, there is some evidence that factories can lead to the clustering of co-regulated genes (**Figures 2 and 3B**). In terms of chromatin structure, there is also some evidence that factories can lead to the formation of chromatin loops (Marenduzzo et al., 2007; Cook, 2010; **Figure 3C**).

Two mechanisms have been suggested by which factories could drive chromatin loop formation (Cook, 2010). First, loops can form when two different genes on the same chromosome associate with the same factory (**Figure 3C**). This could be a chance occurrence, or if specialized factories for certain transcription factors exist, then it could arise because the two genes are both regulated by the same transcription factor(s). There is evidence that such specialized factories do exist (see below), giving rise to speculations that interaction among the shared transcription factors could be involved in recruiting the genes to the same factory (Schoenfelder et al., 2010). In any case, the association of two genes on the same chromosome with the same transcription factory will produce a loop (**Figure 3C**).

A second reason that transcription factories could induce loop formation is the consequence of a physical phenomenon known as the “depletion attraction”. This occurs when larger objects (such as large transcription complexes) are in a crowded environment containing many smaller, soluble objects (such as proteins). The larger objects will tend to aggregate because their clustering excludes the smaller objects from the region of overlap, and this reduces the entropy of the system as a whole (Marenduzzo et al., 2006b). Thus two large polymerase complexes attached to a chromatin fiber would be expected to cluster based on this depletion attraction, thereby contributing to incipient factory formation and in the process producing a loop of chromatin in between the two polymerases (Marenduzzo et al., 2006a, 2007).

There is now experimental evidence from various sources that chromatin loops exist and in fact are commonly found within nuclei. Such loops were initially visualized by light microscopy of lampbrush chromosomes isolated from oocytes of many animals (but not mammals; for review see Morgan, 2002), and by electron microscopy of HeLa cell chromosomes whose nuclei were lysed in non-physiological buffers (Jackson et al., 1984a). In recent years numerous studies using 3C or its derivatives (Tolhuis et al., 2002; Simonis and de Laat, 2008; Schoenfelder et al., 2010) have provided very convincing evidence for chromatin looping in a variety of eukaryotic systems. In a number of cases (e.g., Carter et al., 2002; Tolhuis et al., 2002; Osborne et al., 2004; Spilianakis and Flavell, 2004), widely spaced sequences on the same chromosome are found to be in close contact by 3C, implying the existence of chromatin loops. However, these data by themselves do not demonstrate that transcription factories are the organizing features at the base of those loops. A more direct technique to demonstrate the formation of chromatin loops at RNA polymerase II transcription units is e4C (enhanced ChIP-4C). 4C is 3C on a chip, thereby enabling a genome-wide search for sequences in contact with a given locus. The enhanced form of 4C (e4C) developed by Schoenfelder et al. (2010) incorporates an immunoprecipitation step to enrich for DNA associated with the initiating and elongating form of RNA polymerase II. The authors used the e4C method to study gene–gene associations of mouse globin genes, and found extensive interactions with hundreds of other active genes at transcription factories. These data suggest more directly that loop formation can originate from transcription factories.

Concomitant with a role in chromatin looping, factories may also be involved in clustering of genes in the nucleus. A simple calculation suggests in fact that genes are likely to share factories (**Figure 3C**) since there are not enough factories per cell to provide every active gene with its own factory. Specifically, factory numbers per nucleus range from a few hundred to a few thousand at most, whereas in the human genome, there are currently ~21,000 protein-coding genes and about 12,000 RNA genes known (Flicek et al., 2012) and the number of non-protein-coding genes being identified is still growing. More generally, it is now estimated that ~75–90% of the genes in many different eukaryotic genomes are being expressed based on deep sequencing of entire transcriptomes (Wang et al., 2009). Although the exact number of transcription factories in a cell is still uncertain, and most active genes are not being transcribed continuously but rather in bursts (Chubb et al., 2006; Raj et al., 2006) and it is further not clear that all of these genes are being transcribed in every cell, it seems likely based on this arithmetic that more than one gene is typically transcribed at a given transcription factory.

Direct experimental proof for this possibility has come from a study in mouse erythroid cells. Osborne et al. (2004) used FISH and 3C to show that transcriptionally active genes that were located either megabases apart on the same chromosome or on different chromosomes (*Eraf*, *Hbb-b1*, *Uros*, *Igf2*, *Kcnq1ot1*, and *Hba*) were frequently spatially associated. They found that virtually all (~90%) active alleles of these genes were associated with distinct RNA polymerase II foci, as demonstrated by RNA FISH signals that co-localized with an antibody against initiating and elongating RNA polymerase II phosphorylated at Ser5 on the C terminal domain (CTD). In contrast to active alleles, they found that otherwise identical but inactive alleles in the same cells were repositioned away from transcription factories. Finally, they showed in a triple-labeling experiment that when transcripts from two different genes were co-localized they were often associated with the same RNA polymerase II factory.

In a follow-up study Osborne et al. (2007) investigated the association of another set of genes with transcription factories, in this case during immediate early gene induction in mouse B cells. They found that upon activation the proto-oncogenes *Myc* and *Fos* rapidly relocated to pre-assembled transcription factories and preferentially co-localized with the highly active immunoglobulin heavy chain (*Igh*) locus (Osborne et al., 2007). Before induction most of the alleles of these genes were located away from transcription factories, suggesting that upon stimulus they were actively recruited, which involved a shift in position on average of about 0.5 μm.

## FACTORY SPECIALIZATION

As already noted, the different RNA polymerase forms, I, II, and III, are known to associate in spatially distinct factories specific to each form. There is, however, some evidence for even greater specialization in transcription factories, namely that some factories are specialized for transcription of related genes. For example, such a specialized factory may exist for globin-related genes which may be nucleated by the β-globin locus control region (LCR; Osborne et al., 2004; Ragoczy et al., 2006; Xu and Cook, 2008). In mouse, the LCR is located on chromosome 7 about 60 kb upstream of the *Hbb-b1* (β-globin) gene, which – when activated – contacts the LCR and forms an active chromatin hub (ACH) in a tissue-specific manner (Carter et al., 2002; Tolhuis et al., 2002). Furthermore, *Eraf*, a gene encoding an α-globin-stabilizing protein, which is located ~25 Mb apart from *Hbb-b1*, as well as other erythroid specific active genes are in frequent contact with this ACH (Osborne et al., 2004), suggesting that this ACH is a factory specialized in transcribing globin-related genes.

The hypothesis of specialized transcription factories was experimentally tested by Xu and Cook (2008) who co-transfected monkey COS-7 cells with a number of different mini chromosomes (autonomous replicating plasmids) carrying several different genes controlled by different promoters. After 24 h, there were >8,000 minichromosomes per cell, yet DNA FISH showed that these were concentrated in only ~20 foci. Interestingly, transcripts derived from different types of co-transfected minichromosomes were generally found in distinct foci: minichromosomes with identical promoters tended to cluster at the same factory; furthermore, minichromosomes with introns tended to be transcribed at one set of factories, and minichromosomes without introns tended to be transcribed at another set of factories. This suggests that different types of templates are transcribed in separate factories that specialize in transcribing a certain type of gene or genes (Xu and Cook, 2008).

Further evidence for this possibility comes from Schoenfelder et al. (2010) who observed in mouse erythroid cells that genes regulated by the same transcription factor tended to co-associate at the same transcription factory. Specifically, they found by immunofluorescence that the erythroid transcription factor, KLF1, was present at ~40 discrete foci in the nucleus with nearly all co-localized with active transcription factories, as detected by immunofluorescence with a second antibody against polymerase II phosphorylated at Ser5. This co-localization of transcription factors and factories suggests that there could be “KLF1-preferred transcription factories” (**Figure 3B**), which account for about 10–20% of all factories in that cell type. Consistent with this, Schoenfelder et al. (2010) found that 59–72% of active alleles of KLF1-regulated genes (*Hbb*, *Hba*, *Hmbs*, and *Epb4.9*) were transcribed at KLF1-enriched factories. These association frequencies far exceeded the 10–20% association rate that would be expected if these genes were randomly associating with factories. The authors also found that active pairs of KLF1-regulated genes were co-associated with KLF1 transcription factories at unexpectedly high frequencies (63–79%), although this high degree of co-localization might be largely explained by the tendency of individual KLF1-regulated genes to associate with KLF1 factories, thereby increasing the odds that pairs of co-regulated genes could be at the same factory.

Finally, this clustering of KLF1-regulated genes was itself dependent on KLF1, since KLF1 knockdown disrupted the co-association. Nevertheless, it was also shown that the KLF1 transcription factories are not absolutely required for transcribing KLF1-regulated genes, since transcription of these genes can also occur at other factories lacking an accumulation of KLF1. The authors propose that specialized factories may enhance the expression of clustered and co-regulated genes by placing them in an environment where all the required factors for increased transcription are concentrated.

In their 4C study in mouse erythroid cells, Simonis et al. (2006) also examined one of the KLF1-regulated genes, namely *Hbb*, but in contrast to Schoenfelder et al. (2010) they found no enrichment for erythroid specific genes associated with *Hbb*. Although Schoenfelder et al. (2010) used e4C while Simonis et al. (2006) used 4C, this is unlikely to explain the difference in results since in these mouse erythroid cells 90% of the *Hbb* alleles are active (Osborne et al., 2004) and therefore 4C and e4C should yield comparable contact partners. Thus further work will be necessary to understand this disparity. Another potential difference with the KLF1 results comes from the analysis of polymerase factories at a tandem array containing multiple promoter targets for the glucocorticoid receptor (GR) (Müller et al., 2007). At this array, clusters of GR did not directly co-localize with polymerase factories in contrast to the direct overlap of KLF1 clusters and polymerase factories seen in mouse erythroid cells. This difference could reflect the different transcription factors under study, or it could also reflect the artificial nature of the tandem array.

In summary the results of some studies, suggest that “specialized” transcription factories may exist (**Figure 3B**). However, other studies suggest this is not always the case, indicating that “specialized” transcription factories may be cell type- and transcription factor-dependent.

## FUNCTIONAL CONSEQUENCES OF TRANSCRIPTION-FACTORY INDUCED GENE CLUSTERING

It is possible that gene clustering at factories simply reflects chance interactions imposed by the requirement to undergo transcription at a limited number of sites, but it is also possible that clustering of genes at factories has functional consequences. Indeed, the rationale behind building any sort of factory is “to enhance production by concentrating the relevant machines, resources, and expertise in one place” (Papantonis and Cook, 2011). This suggests that one functional consequence of transcription factories could be increased efficiency in transcription. This is in fact the idea behind the proposal noted above that at specialized transcription factories many of the co-regulatory molecules normally associated with a specific transcription factor become concentrated at the transcription factory site leading to more efficient transcription of co-regulated genes that are clustered at that factory. Although this is an attractive idea, it is difficult to test directly, as that requires preventing certain genes from associating with certain factories, and at the moment it is not clear how to accomplish this. Indeed, an understanding of the functional consequences of gene–gene association at factories requires first a better understanding of the mechanisms that lead to this association before it will be possible to disrupt these mechanisms and then assay the consequences on transcription.

In addition to increased transcription efficiency, another potential functional consequence of gene–gene associations at transcription factories could be to inadvertently facilitate gene translocation events that can lead to malignant transformations. For example, *Myc* and *Igh* are the most common translocation partners in plastocytoma and Burkitt lymphoma, and as noted above these two loci frequently co-localize (Roix et al., 2003) in B cells, at least in part because they share the same transcription factory (Osborne et al., 2007). A similar argument has been made for translocation events between the mixed lineage leukemia locus (MLL) and the *AF4* and *AF9* genes (Cowell et al., 2012). Such translocations arise in therapy-related acute myeloid leukemias, which are secondary cancers caused by certain forms of chemotherapy, specifically alkylating agents or topoisomerase poisons. Cowell et al. (2012) found that *AF4* and *AF9* were more frequently associated with MLL in the same transcription factory compared to several other genes that did not show high translocation frequencies with MLL. They suggest that this proximity coupled with the DNA strand breaks induced by the topoisomerase poison can lead to these preferred translocation events and thereby cause therapy-related acute myeloid leukemias. However, a recent study has argued that recurrent translocation frequencies are not correlated with the rate of gene:gene interactions *per se*, but instead with the rates of DNA damage at different loci (Hakim et al., 2012). Nevertheless, translocation requires that two genes be near each other, and so it will be interesting to determine in future studies if translocation events between genes occur more frequently when the two genes are in the same factory, as the act of transcription itself may make genes more susceptible to DNA damage.

## ALTERNATIVE MODELS FOR GENE–GENE CLUSTERING IN NUCLEI

**FIGURE 4:**
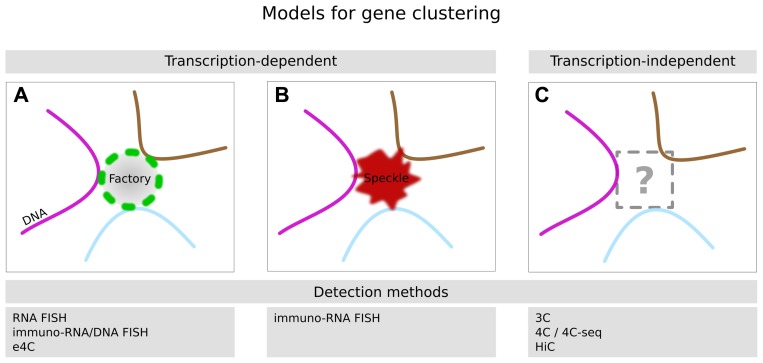
**Potential mechanisms of gene clustering.** Genes can be found in close proximity inside the nucleus even though the corresponding genomic loci may not even be on the same chromosome, or if they are on the same chromosome they may be located megabases apart. **(A)** Some evidence suggests that gene clustering occurs by sharing of the same transcription factory: the limited number of available transcription factories implies that multiple active genes (three are shown) are transcribed at the same factory. In special cases a sub-population of factories might be specialized in transcribing groups of genes that are regulated by a certain transcription factor (i.e., KLF1). These factories are then enriched with the transcription factor and lead to the formation of co-regulated gene clusters. **(B)** An alternative model for cluster formation is the co-association of multiple genes with the same SC35 splicing speckle, which are also limited in number in a given nucleus. **(C)** Genes can also cluster in the absence of transcription or splicing and are organized in a pre-established architecture which is not influenced by transcriptional activation or repression. The organizing principle(s) for this third type of clustering remains to be identified, but may include preferential positioning of chromosomes, sharing of nuclear factors other than speckles or factories, or association with nucleoli or components of the nuclear envelope.

While the preceding data implicate a role for transcription factories in gene co-localization (**Figure 4A**), data from other groups suggest several alternative mechanisms for such co-localization. For example, by combining immunofluorescence with DNA FISH, a number of studies have suggested that SC35 splicing speckles could be responsible for gene clustering (Shopland et al., 2003; Brown et al., 2006, 2008; Takizawa et al., 2008; Szczerbal and Bridger, 2010; **Figure 4B**). Splicing speckles are nuclear structures of ~0.5–2 μm in size characterized by an enrichment in the SR splicing factor, which has a critical role in transcription and elongation (Lin et al., 2008). Frequent sharing of the same splicing speckle has been demonstrated in human erythroblast cells for α- and β-globin and other erythroid specific genes (Brown et al., 2006, 2008), as well as in porcine adipocytes for key adipogenic genes (Szczerbal and Bridger, 2010). These various genes are either found megabases apart on the same chromosome or on different chromosomes, so their co-association with each other and with the same splicing speckle suggests that they could be brought together by their common need for splicing components found in the speckle. This could also in part explain the observation that minichromosomes carrying introns tend to cluster in nuclear foci distinct from foci with minichromosomes that lack introns (see above; Xu and Cook, 2008).

However, it is important to point out that not all cells exhibit speckles. For example, Schoenfelder et al. (2010) found that speckles were absent in fetal *ex vivo* mouse erythroid cells, and Saitoh et al. (2012) found that nuclear speckles were found in only certain cell types in the mouse. Together these observations indicate that speckles cannot be universally responsible for gene–gene clustering, since they are not present in all cell types.

Indeed, other data suggest that some genes can cluster without sharing either speckles or factories. Specifically, some studies suggest that certain sets of genes can be co-localized even in the absence of transcription (**Figure 4C**). Simonis et al. (2006) used 4C to show that the active β-globin locus in murine fetal liver is interacting with many (20–50) other active genes. Then, in a succeeding study, they inhibited transcription using either DRB or α-amanitin, and showed that the great majority of long-range intra- or inter-chromosomal interactions of the β-globin locus remained unaltered (Palstra et al., 2008), even though RNA polymerase II was no longer detectable by ChIP within the β-globin locus. They observed a similar result for the ubiquitously expressed *Rad23* gene, suggesting that ongoing transcription or polymerase binding is not essential for maintaining some of the global genome organization in a cell. Note, however, that these results do not exclude the possibility that RNA polymerase II had a role in establishing this organization before transcription was blocked.

Some other studies have also hinted at the possibility of transcription-independent gene clustering. In a recent study of GR responsive loci, the authors show that genes which are either induced or repressed by GR are already clustered before hormone induction of GR. Further, the relative positions of induced and repressed genes did not change dramatically following hormone treatment and transcriptional induction (Hakim et al., 2011). A similar conclusion was also drawn by another group studying estrogen-receptor responsive loci before and after stimulation by hormone (Kocanova et al., 2010). Thus, together these data from several different systems raise the possibility that in some cases the overall spatial configuration of genes can be pre-established and neither transcriptional activation nor inhibition disturbs this configuration, at least not enough to be detected by 3C or 4C (**Figure 4C**). It remains uncertain what might specify this global organization, but several possibilities have been suggested, namely nucleosomal positioning, histone modifications, or transcription factor binding (Schübeler et al., 2000; Palstra et al., 2008; Hakim et al., 2011). One possible explanation for this phenomenon in terms of the factory model for genome organization is that some inducible genes might already be prepositioned at poised factories in order to quickly respond to gene activation.

Finally, when considering how gene–gene associations may occur in the nucleus, it is important to keep in mind that multiple factors regulate the spatial organization of the genome. These include the clustering of active and inactive genes within the linear DNA sequence, the organization of chromosomes into territories which may preferentially associate with other territories and rapidly rearrange in response to some stimuli (Mehta et al., 2010), the association of some genes with specific DNA-organized compartments, such as nucleoli, the association of some activated genes in yeast with nuclear pores, and the association of some repressed genes in mammalian cells with the nuclear lamina (reviewed in Fedorova and Zink, 2009; Taddei et al., 2010). Some or all of these factors are likely to contribute, along with transcription factories and splicing speckles, to the final configuration of genes within each nucleus.

Thus, due to a combination of some of the preceding factors, certain genes may already be near each other before transcription is induced. As transcription begins these genes may move randomly to the nearest factory and/or speckle leading to further co-localization. Some evidence in support of this combination of clustering mechanisms comes from a recent study of co-regulated genes involved in adipogenesis. Here it was found that both polymerase factories and speckles contributed to clustering of the co-regulated genes, with the role of each in clustering proportional to the average number of factories or speckles in the nuclei of the cells under study (Rieder et al., in revision).

## CONCLUSIONS

After two decades of work there is now considerable evidence in many different cell types confirming the existence of transcription factories. However, several corollaries of the basic transcription factory model remain uncertain. While there is accumulating evidence that the DNA template is drawn through a stationary factory, more direct demonstrations of this process will be required before student text books are re-written. Furthermore, the importance of transcription factories as organizers of nuclear architecture remains to be fully understood. Specifically, it remains uncertain whether factories are the central organizing principle or instead one of many factors that contribute to gene clustering. In addition, more data and experiments are needed to assess a possible role of transcription factories in cancer facilitating chromosome translocation or other types of disease. Progress in deciphering the role of factories in nuclear organization will certainly come from continued application of next-generation sequencing methods to specialized derivatives of 3C. High throughput microscopy should also enable analysis of a much larger number of genomic loci leading to more general insights into the role of transcription in the spatial organization of the genome. Finally, further elucidation of transcription factory structure, including analysis within live cells, will benefit considerably from the emergence of super-resolution microscopy.

## Conflict of Interest Statement

The authors declare that the research was conducted in the absence of any commercial or financial relationships that could be construed as a potential conflict of interest.
